# Supplementary material for: An integrative multiomic network model links lipid metabolism to glucose regulation in coronary artery disease
Source: Nat Commun. 2021 Jan 22;12:547. doi: 10.1038/s41467-020-20750-8 (PMC7822923; doi:10.1038/s41467-020-20750-8)
Supplement: Supplementary file 1 — Supplementary Information [file 41467_2020_20750_MOESM1_ESM.pdf]

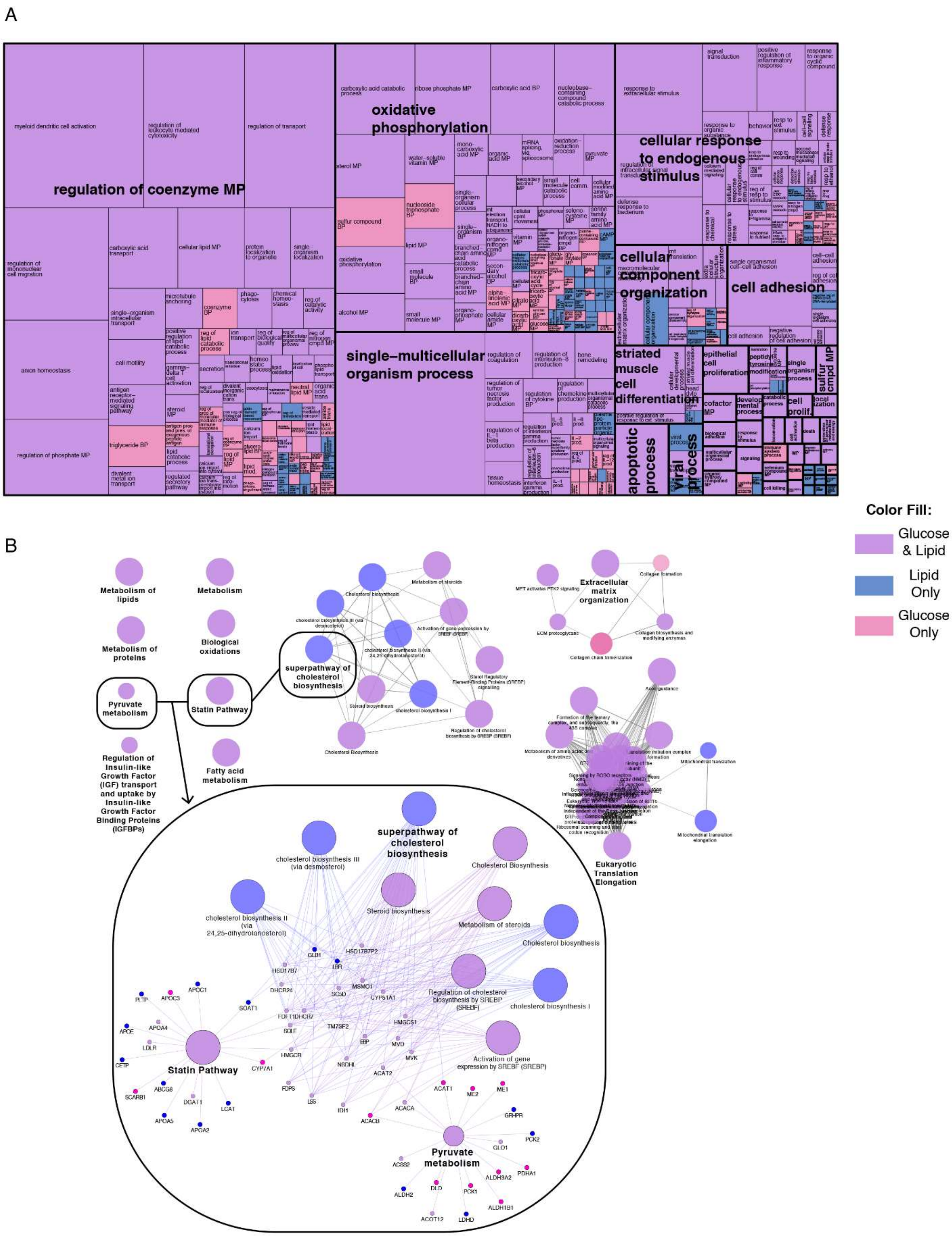
A
regulation of coenzyme MP
oxidative phosphorylation
cellular response to endogenous stimulus
single−multicellular organism process
cellular component organization
cell adhesion
striated muscle cell differentiation
apoptotic process
viral process
myeloid dendritic cell activation
regulation of leukocyte mediated cytotoxicity
regulation of transport
regulation of mononuclear cell migration
anion homeostasis
regulation of phosphate MP
triglyceride BP
sulfur compound BP
Color Fill:
Glucose & Lipid
Lipid Only
Glucose Only
B
Metabolism of lipids
Metabolism
Metabolism of proteins
Biological oxidations
Pyruvate metabolism
Statin Pathway
superpathway of cholesterol biosynthesis
Regulation of Insulin-like Growth Factor (IGF) transport and uptake by Insulin-like Growth Factor Binding Proteins (IGFBPs)
Fatty acid metabolism
Extracellular matrix organization
Eukaryotic Translation Elongation
cholesterol biosynthesis III (via desmosterol)
Cholesterol Biosynthesis
cholesterol biosynthesis II (via 24,25-dihydrolanosterol)
Steroid biosynthesis
Metabolism of steroids
Cholesterol biosynthesis
Regulation of cholesterol biosynthesis by SREBP (SREBF)
cholesterol biosynthesis I
Activation of gene expression by SREBF (SREBP)
Pyruvate metabolism

Supplementary Fig. 1. Gene Ontology analysis. (A) Gene Ontology (GO) enrichment analysis REVIGO[71] word plot for all trait correlated DE genes in all seven tissues for lipid related traits and glucose related traits. GO terms are collapsed into grouping based on GO hierarchy. Color represents if the GO term was shared between lipid and glucose traits or was unique. The size of each box is proportional to the number of times it was found as a GO term. (Abbreviations in the text are MP: metabolic process, BP: biosynthetic process). (B) GO network plot for Liver trait correlated DE genes for lipid traits (LDL, HDL, plasma cholesterol, hyperlipidemia, cholesterol medications) and glucose traits (blood glucose, HbA1c, T2D status, T2D medications, insulin medications). Nodes are colored based on the sharing of pathways between the two lists.

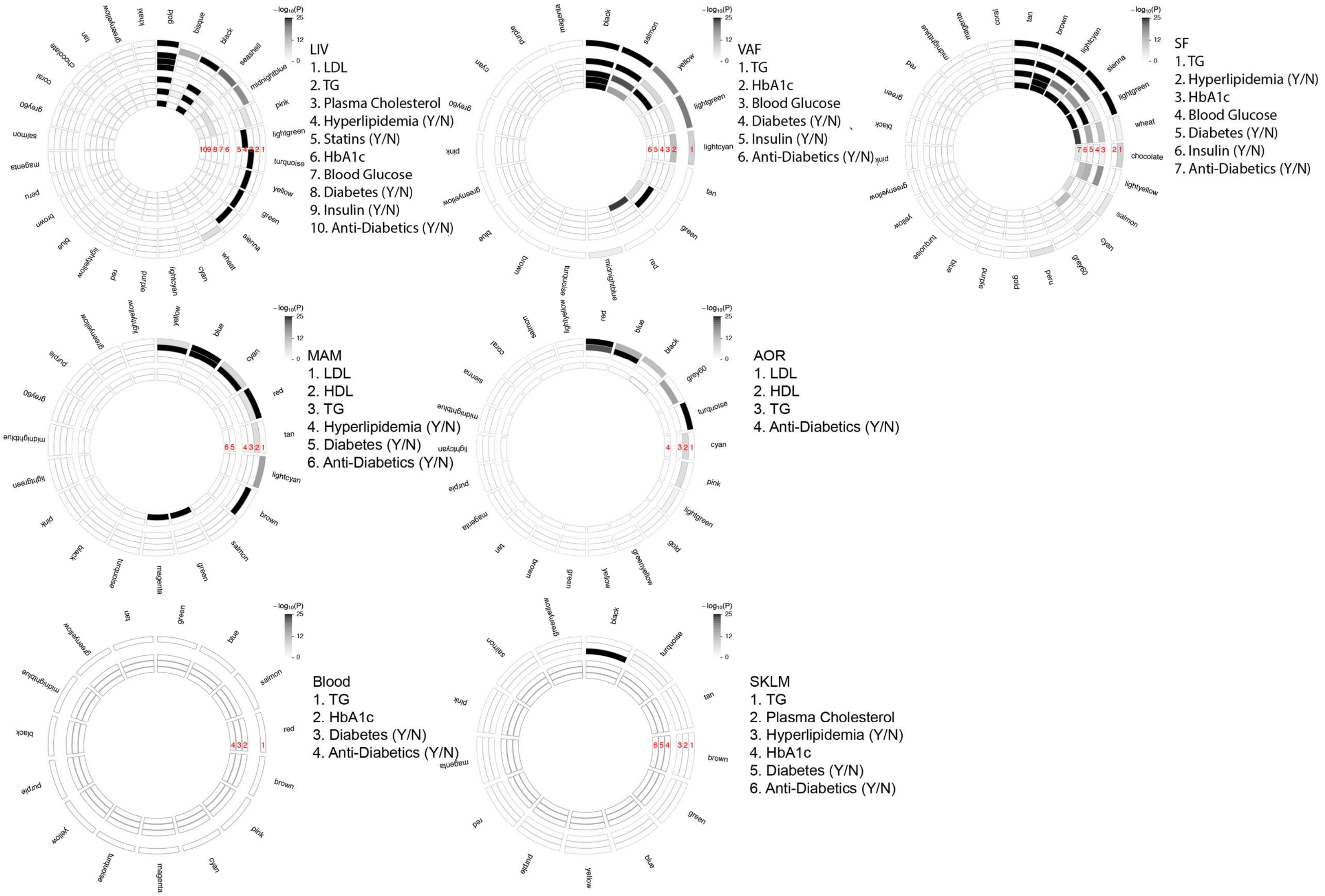
LIV
1. LDL
2. TG
3. Plasma Cholesterol
4. Hyperlipidemia (Y/N)
5. Statins (Y/N)
6. HbA1c
7. Blood Glucose
8. Diabetes (Y/N)
9. Insulin (Y/N)
10. Anti-Diabetics (Y/N)
VAF
1. TG
2. HbA1c
3. Blood Glucose
4. Diabetes (Y/N)
5. Insulin (Y/N)
6. Anti-Diabetics (Y/N)
SF
1. TG
2. Hyperlipidemia (Y/N)
3. HbA1c
4. Blood Glucose
5. Diabetes (Y/N)
6. Insulin (Y/N)
7. Anti-Diabetics (Y/N)
MAM
1. LDL
2. HDL
3. TG
4. Hyperlipidemia (Y/N)
5. Diabetes (Y/N)
6. Anti-Diabetics (Y/N)
AOR
1. LDL
2. HDL
3. TG
4. Anti-Diabetics (Y/N)
Blood
1. TG
2. HbA1c
3. Diabetes (Y/N)
4. Anti-Diabetics (Y/N)
SKLM
1. TG
2. Plasma Cholesterol
3. Hyperlipidemia (Y/N)
4. HbA1c
5. Diabetes (Y/N)
6. Anti-Diabetics (Y/N)

Supplementary Fig. 2. Differential expression circular plots for all tissues. Enrichment of genes for every tissues' coexpression modules with the trait correlated DE genes. Each track represents a different phenotype and the fill represents the $-\log_{10}$(Fisher's Exact Test *P*-value). Only those with an FDR <= 5% are shown.

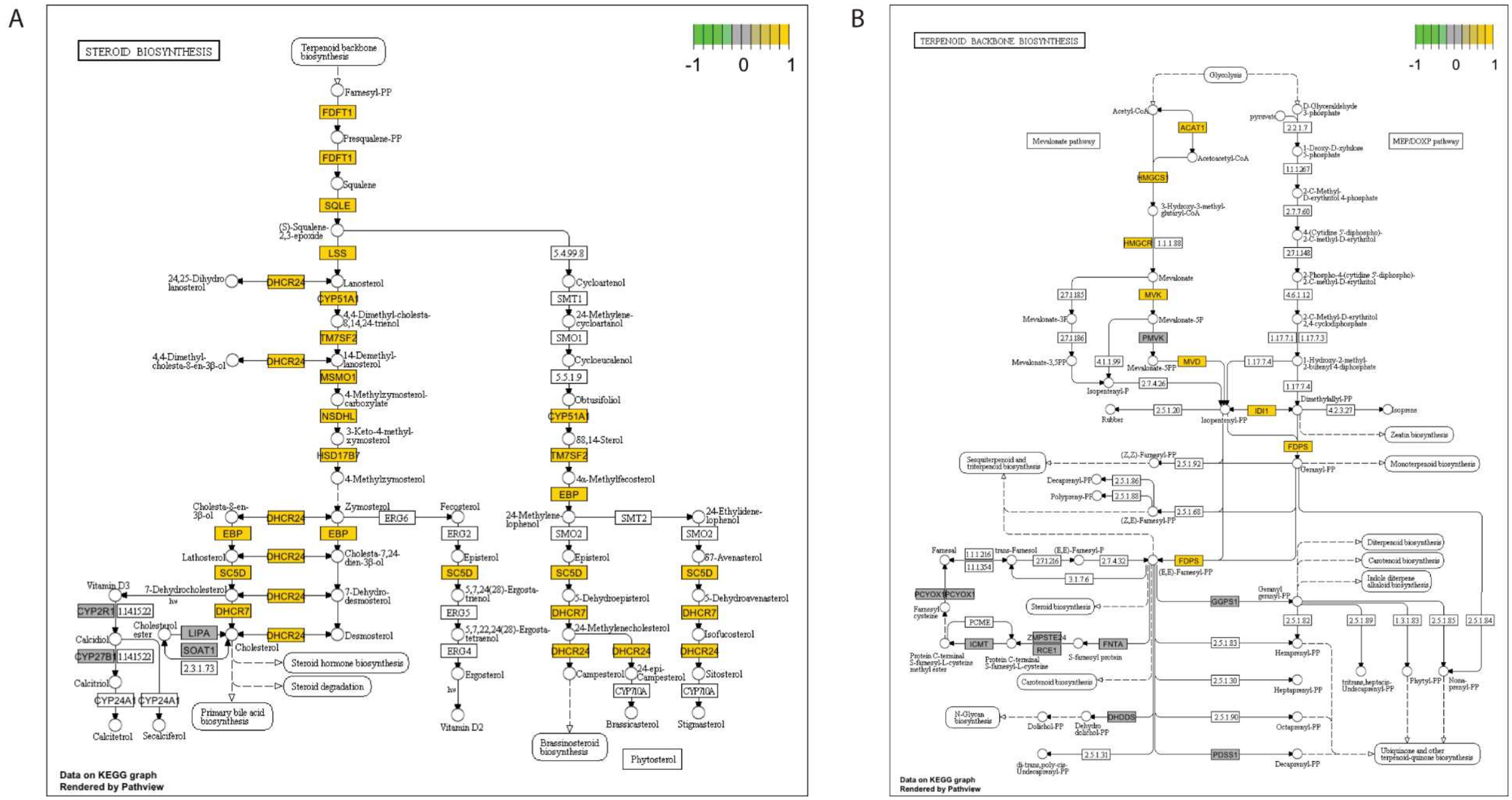

Supplementary Fig. 3. **KEGG pathways enriched for the GLD module genes.** Left panel: Sterol Bioynthesis; right panel: Terpenoid Backbone Biosynthesis. Genes in yellow are the genes found in the GLD module while the genes colored in grey are those, which were not found in the GLD module but expressed in the data.

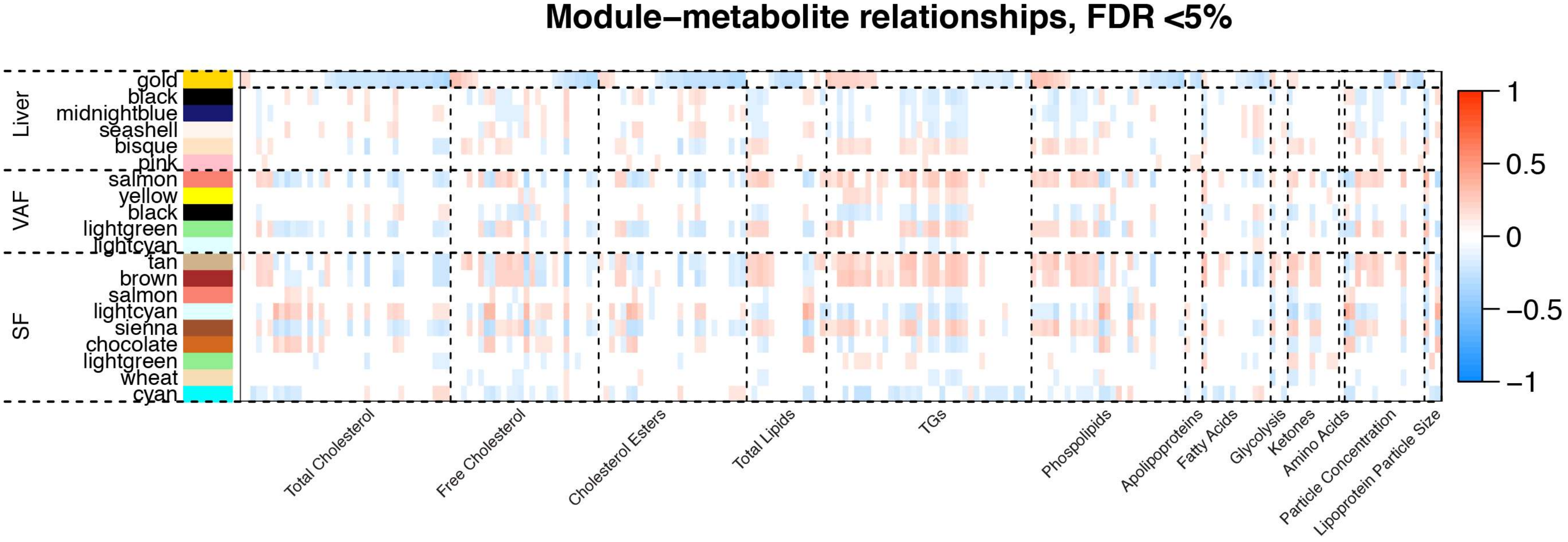

Supplementary Fig. 4. Metabolite – module correlation for the 20 modules in Liver, VAF and SF. Only shown are the correlation r value where the FDR < 5%. Metabolites are clustered by group type.

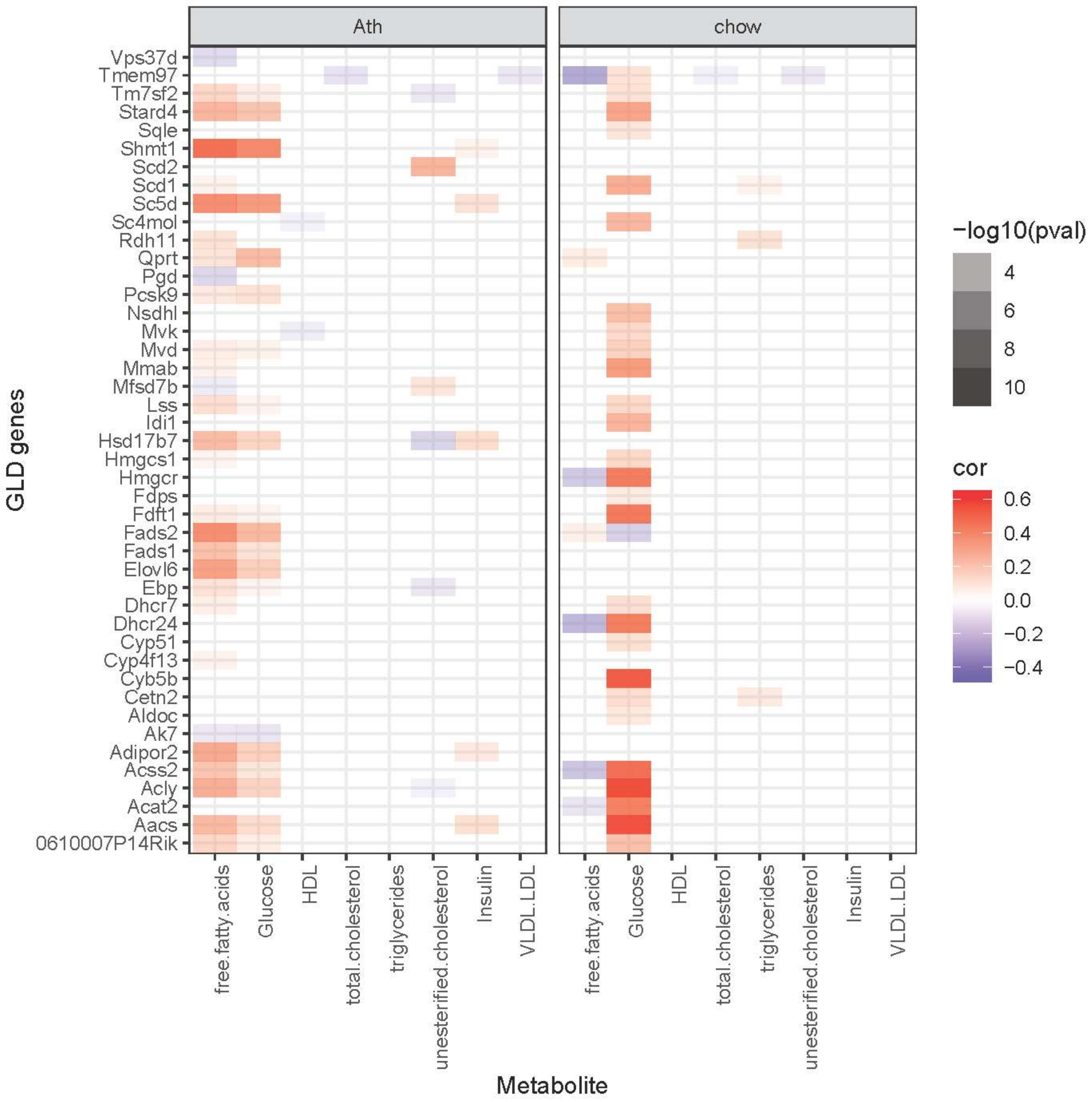

Supplementary Fig. 5. **Correlations between GLD module genes and metabolite levels in the Hybrid Mouse Diversity Panel (HMDP) dataset.** Both in mice fed an Apoe Leiden diet (a model of atherosclerosis, labeled "Ath") and mice fed a normal chow diet ("chow"), GLD module genes are significantly associated with both blood glucose levels and blood lipid levels.

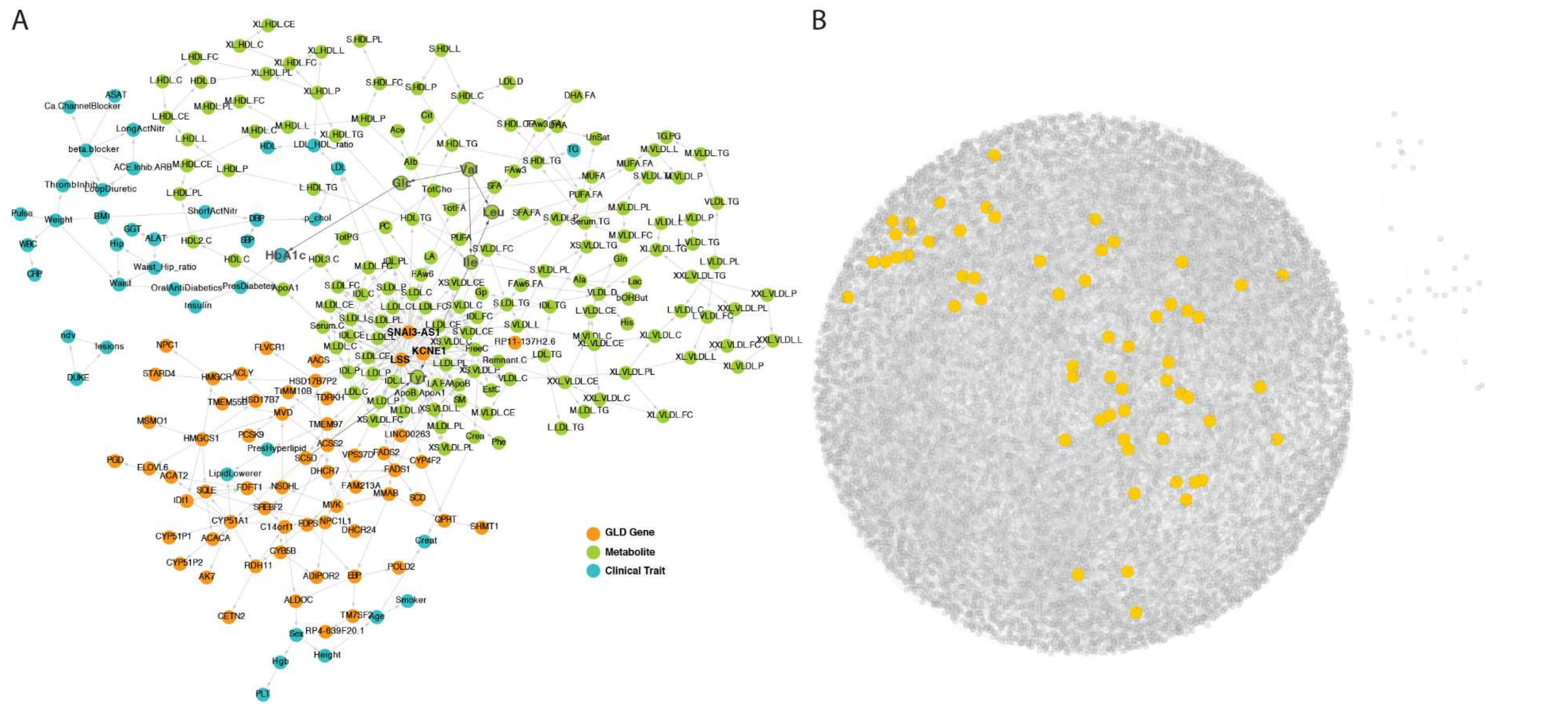

Supplementary Fig. 6. Additional probabalistic causal network diagrams. (A) Multiscale (MS) probabilistic causal network for the GLD module genes, metabolites, and clinical traits. Color represents the data source and genes in bolded black represent the key drivers of the network. Dark grey edges and nodes are highlighted to show the interaction between the GLD genes and glucose via amino acids. (B) Global network (N = 8812) all genes are in grey except for GLD module genes, which are colored in gold and enlarged. The networks are laid out in spring-weighted measure where the weight is the edge-betweenness, a metric of how much information flows through that edge.

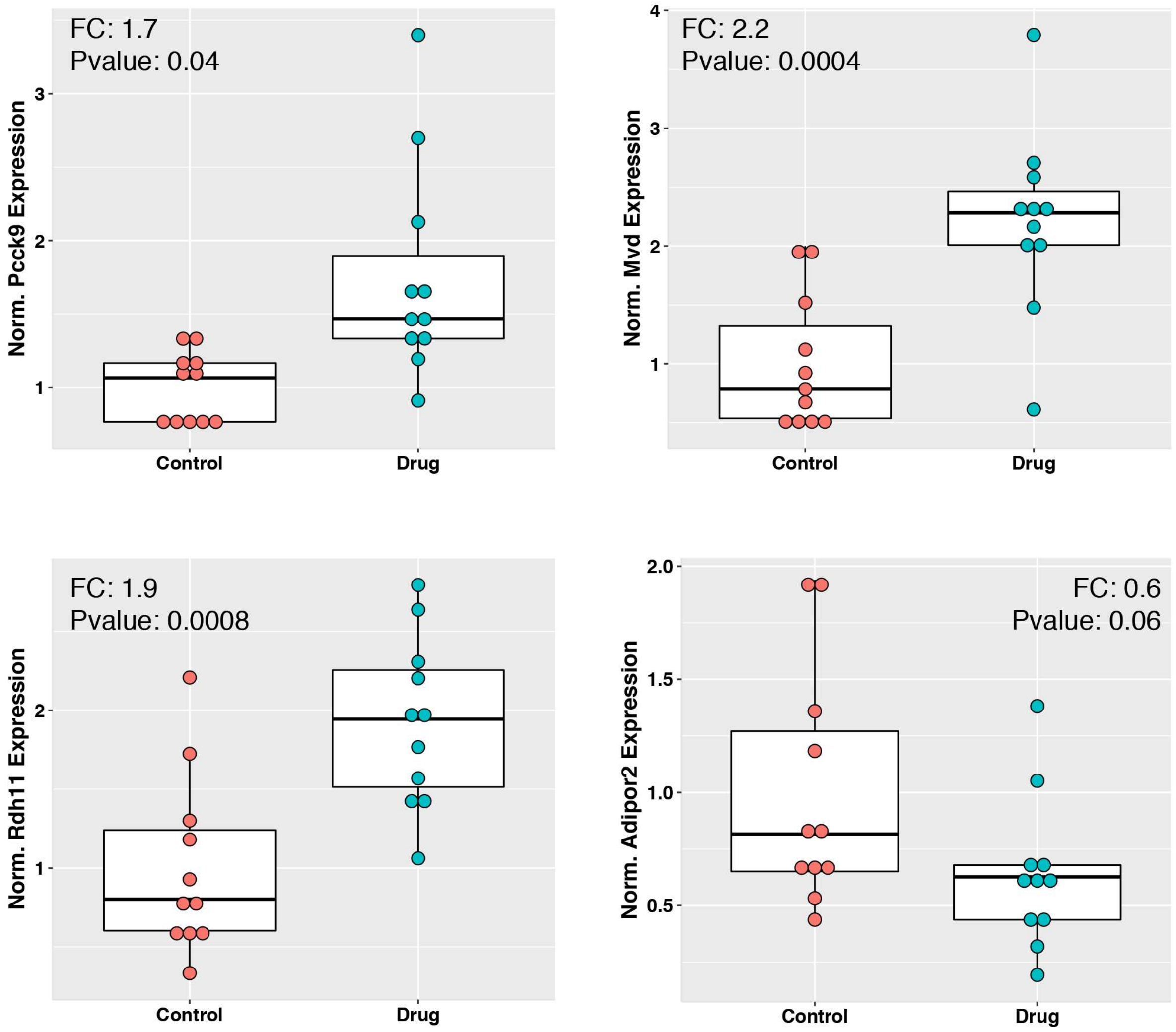

Supplementary Fig. 7. Gene expression data from mouse experiment. Boxplots for the qPCR results from the liver expression of the B6 mice fed either control diet (n = 11 animals) or BIBB515 diet (n = 11 animals) are shown for the genes of Psck9, Mvd, Rdh11 and Adipor2. Center line, median; box limits, upper and lower quartiles; whiskers, 1.5x interquartile range; points, all individual data points. *P*-values calculated with Student's t-test, two-sided, with no correction for multiple hypothesis testing applied.

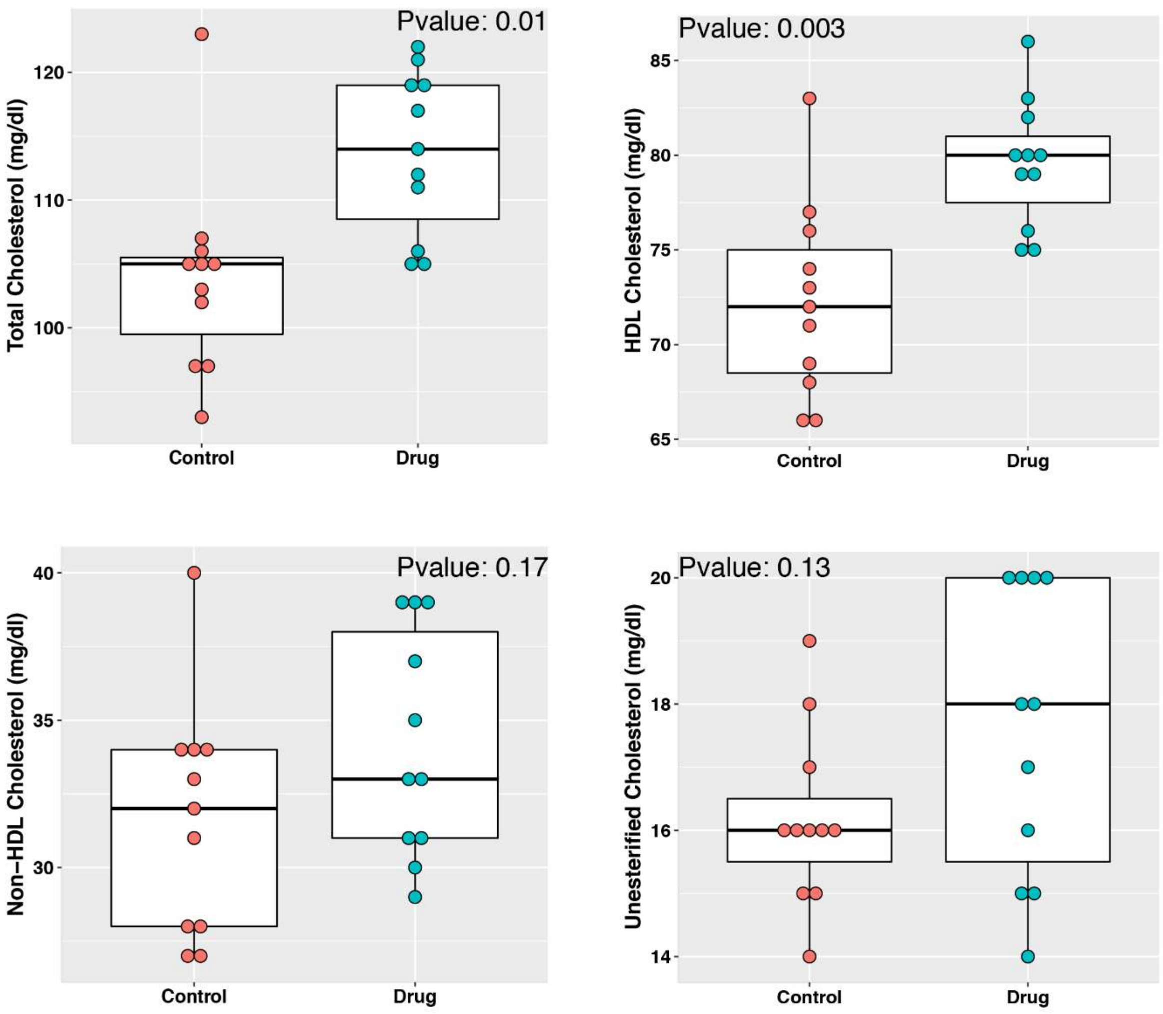

Supplementary Fig. 8. Cholesterol results from mouse experiment. Boxplot for the cholesterol results from the mice B6 blood samples after being fed either control diet (n = 11 animals) or BIBB515 diet (n = 11 animals). Center line, median; box limits, upper and lower quartiles; whiskers, 1.5x interquartile range; points, all individual data points. *P*-values calculated with Student's t-test, two-sided, with no correction for multiple hypothesis testing applied.

Supplementary Table 1.

The 20 modules enriched for both glucose and lipid correlated DE genes, and the variance explained by their 1st PC.

Module	Tissue	# Lipid DE traits enriched for	# Gluc DE traits enriched for	Variance Explained by Module PC1	Module Lipid Correlation (direction)	Module Glucose Correlation (direction)
gold	LIV	4	3	57%	LDL (-), Plasma Cholesterol (-)	HbA1c (+), Blood Glucose (+)
black	LIV	1	3	45%	TG (-)	HbA1c (-)
midnightblue	LIV	1	2	59%	TG (-)	
seashell	LIV	1	3	57%	TG (-)	HbA1c (-)
bisque	LIV	1	3	57%	TG (+)	HbA1c (+)
pink	LIV	1	2	49%		
salmon	VAF	1	3	53%	TG (+)	HbA1c (+), Blood Glucose (+)
yellow	VAF	1	3	45%	TG (+)	HbA1c (+)
black	VAF	1	4	46%	TG (-)	HbA1c (-), Blood Glucose (-)
lightgreen	VAF	1	2	51%	TG (+)	HbA1c (+), Blood Glucose (+)
lightcyan	VAF	1	1	59%	TG (-)	HbA1c (-)
tan	SF	1	4	49%	TG (+)	HbA1c (+)
brown	SF	1	4	42%	TG (+)	HbA1c (+)
salmon	SF	1	2	51%	TG (-)	
lightcyan	SF	1	4	50%	TG (-)	HbA1c (-)
sienna	SF	1	3	50%	TG (+)	HbA1c (+)
chocolate	SF	1	3	54%	TG (-)	
lightgreen	SF	1	3	64%	TG (+)	HbA1c (+)
wheat	SF	1	3	59%		HbA1c (-)
cyan	SF	1	1	47%	HDL (-)	
peru	SF	1	0	55%	TG (+)	HbA1c (+)
brown	MAM	1	0	42%	HDL (-)	Blood Glucose (-)

Supplementary Table 2
Correlation statistics of the 1st PC of the GLD-equivalent module (bisque) in the obese cohort liver with glucose and lipid trait data available in that cohort.

**Trait**	**Correlation (Pearson's r)**	**FDR**
Blood Insulin	0.094	0.036
Blood Glucose	0.13	0.0053
HbA1c	0.093	0.091
LDL Cholesterol	-0.16489	0.0011
HDL Cholesterol	-0.05046	0.453061
Triglycerides	0.243345	1.35E-08
Plasma Cholesterol	-0.01145	0.838924

Supplementary Table 3.
Enrichment analysis results from GTEx tissues.

Tissue	OR	FDR	Module Size	# Gold Genes in Module	Age
**Liver**	3368.10	1.86E-88	47	35	Old
**Liver**	5172.02	1.15E-77	36	30	Young
**Esophagus Mucosa**	2240.90	2.35E-66	38	27	Old
**Cells Transformed fibroblasts**	838.24	3.10E-46	39	21	Young
**Lung**	465.97	1.73E-28	37	14	Young
**Cells Transformed fibroblasts**	63.43	1.79E-27	280	22	Old
**Artery Aorta**	91.17	3.15E-21	129	15	Old
**Lung**	31.30	7.28E-13	325	13	Old
**Esophagus Mucosa**	6.73	5.99E-08	2374	21	Young
**Thyroid**	16.82	1.05E-05	330	8	Young
**Thyroid**	10.51	1.11E-05	674	10	Young
**Nerve Tibial**	34.77	6.14E-05	96	5	Young
**Skin Sun Exposed (Lower leg)**	5.59	7.53E-05	1960	15	Old
**Liver**	31.60	4.88E-04	73	4	Young
**Muscle Skeletal**	14.17	5.31E-04	241	6	Old
**Nerve Tibial**	61.09	1.14E-03	33	3	Young
**Lung**	11.40	1.18E-03	349	6	Old
**Adipose Subcutaneous**	4.86	1.35E-03	1810	13	Old
**Lung**	5.82	1.87E-03	1055	9	Old
**Thyroid**	10.15	2.17E-02	320	5	Old
**Adipose Subcutaneous**	11.64	4.35E-02	207	4	Young
**Adipose Subcutaneous**	16.32	4.44E-02	110	3	Old

Supplementary Table 4.
Liver modules correlated to GLD module at FDR <5% and the number of edges to & from GLD genes in the expanded and global BNs.

Module	FDR	Size	Correlation	# edges Gold -> module in expanded BN (path 1)	# edges module -> gold in expanded BN (path 1)	# edges Gold -> module in global BN (path 1)	# edges module -> gold in global BN (path 1)
gold	0.00E+00	60	1.000	84	84	81	81
bisque	4.42E-20	30	0.404	4	1	4	0
lightgreen	6.05E-09	75	0.267	1	0	0	0
wheat	6.63E-07	44	0.231	0	1	0	0
chocolate	1.67E-06	41	0.118	0	0	0	0
yellow	5.15E-04	595	0.111	3	5	3	3
purple	5.90E-03	134	-0.102	0	0	0	0
peru	1.62E-02	45	-0.118	0	0	0	0
midnightblue	1.62E-02	78	-0.134	0	0	0	0
black	2.45E-02	455	-0.165	0	0	0	0
seashell	4.02E-02	35	-0.223	0	0	0	0

Supplementary Note 1: Code Supplement

All analyses were performed with previously published packages. This document contains details about how we invoked those packages. Unless otherwise noted, these analyses were run in R. See the documentation of these methods for more information.

For information about the sequence of these analyses and the rationale behind them, see the Methods section of the main text.

1 Normalization and Quality Control

Normalization and quality control were performed in R using the `limma`, `edgeR`, and `variancePartition` libraries, all publicly available on Bioconductor. The normalization and quality control pipeline starts with the following input:

- A matrix `data` of expression data (counts) for a single tissue. Rows are transcripts and columns are samples.
- A data frame `info` containing all technical and phenotypic information for each sample. Rows are samples and columns are fields. Make sure the samples are aligned with the columns of `data`.

Each step of this pipeline is run using a single tissue, and repeated for all seven tissues.

1.1 First Pass Filtering and Normalization

We performed Initial steps of filtering and normalization using the `cpm()` and `calcNormFactors()` functions from the `edgeR` package and the `voom()` function from the `limma` package.

```
isexpr = rowSums(cpm(data )>1) >=  .10*ncol(data)

genesAll <- DGEList(counts=data[isexpr,])
genesAll <- calcNormFactors(genesAll)

vobj  = voom(genesAll)

# save filtered and normalized data here
```

1.2 Variance Partition Analysis

We performed variance partition analysis using the `variancePartition` package. Here, `form` is a formula containing the variables to consider; see the package documentation of `variancePartition` for details. `vobj` is the output of `voom()` from step 1.1.

```
varPart = fitExtractVarPartModel( vobj, form, info )

x= plotVarPart(varPart)

# show or save the variance partition plot here
```

1.3 Flow Cell and Hardware Correction

We corrected for flow cell and hardware using the `lmFit()` function from the `limma` package. As described in Methods, we corrected for flow cell for all tissues and additionally corrected for hardware for visceral fat and blood. Note that for this correction to be reliable, each flow cell or hardware must have processed at least about 10 samples. `design` is a design matrix, where columns are flow cell identifiers or hardware identifiers and rows are samples, and each cell contains 1 if that sample was processed on that flow cell or hardware and 0 otherwise. We removed multicolinearity in the design matrix using `svd` before performing the correction. `vobj` is the output of `voom` from step 1.1.

```
svd_design = svd(design)
design_matrix_svd =svd_design$u[,svd_flowCell$d>10^-10]

fit = lmFit(vobj, design_matrix_svd)
resid = residuals(fit, vobj)

# save corrected data here
```

1.4 PCA and Outlier Removal

We used builtin R functions to perform PCA on the normalized and corrected expression data. We then used `ggplot2` to determine outliners, by drawing an ellipse in PC1 vs PC2 space and identifying points outside of that ellipse. Note, this code requires a column name col, corresponding to a field in the `info` data frame, which is only used to color points in the plot; outliers can be computed without specifying this.

```
covariance=cov(resid)
SampleByVariable=t(covariance)
clonename<-rownames(SampleByVariable)
pca <- prcomp(SampleByVariable, scale=T)
summ=summary(pca)
```

```
level3=pnorm(3,mean=0,sd=1,lower.tail=T) - pnorm(3,lower.tail=F)
level2=pnorm(2,mean=0,sd=1,lower.tail=T) - pnorm(2,lower.tail=F)
level1=pnorm(1,mean=0,sd=1,lower.tail=T) - pnorm(1,lower.tail=F)

a <- ggplot(data.frame(pca$x),
                       aes(x= pca$x[,1], y= pca$x[,2],
                       factor(info[,col]), label=clonename)) +
geom_point(size=0.8) +
geom_text(aes(label=clonename), hjust=0, vjust=0, size=1.2) +
labs(title="PC1-PC2") +
xlab(paste("PC1: ",
            round(summ$importance[2,1]*100, digits=2),"%",sep="")) +
ylab(paste("PC2: ",
            round(summ$importance[2,2]*100,digits=2),"%",sep="")) +
ggtitle(paste("Colored By:",col)) +
stat_ellipse(aes(x = pca$x[,1],y=pca$x[,2]),inherit.aes=F,
                type="norm",level=level3,
                linetype = "dotdash", colour="darkgrey") +
stat_ellipse(aes(x = pca$x[,1],y=pca$x[,2]),inherit.aes=F,
                type="norm",level=level2,
                linetype = "dotdash", colour="darkgrey") +
stat_ellipse(aes(x = pca$x[,1],y=pca$x[,2]),inherit.aes=F,
                type="norm",level=level1,
                linetype = "dotdash",colour="darkgrey")
build <- ggplot_build(a)$data
points <- build[[1]]
ellipse <- build[[3]]
dat <- data.frame(points[1:2], in.ellipse = as.logical(
                    point.in.polygon(points$x, points$y,
                                     ellipse$x, ellipse$y)))
outliers=points$label[which(dat$in.ell==F)]

# show or save the PCA plot here

resid = resid[,setdiff(colnames(resid), outliers)]
info = info[match(colnames(resid), info$id),]

# save filtered data here
```

2 Differential Expression

We performed differential expression analysis using the `lmFit()`, `contrast.fit()`, and `eBayes()` functions from the `limma` package.

2.1 Continuous Traits

The code below is applied to each continuous trait in `info` to detect genes whose expression is significantly correlated with that trait. Categorical traits are treated differently, see step 2.2 below. `info` and `resid` are the normalized and filtered data reulting from step 1.4; `col` is the column name.

```
design = model.matrix(~ info[,col])
fit    = lmFit(resid,design)
fit2 <- eBayes(fit)

topSet = topTable(fit2, number=nrow(fit2))

# output or analyze top DE results here
```

2.2 Categorical Traits

The code below is applied to each categorical trait, to detect genes whose expression is significantly different between different categories. `resid`, `info`, and `col` are the same as in step 2.1 above.

```
tmp = info[,col]
design = model.matrix(~ 0 + tmp )
colnames(design)=gsub("tmp","",colnames(design))

levs = colnames(design)
combos = combn(levs,2)
contrasts = apply(combos,2, function(x){paste(sort(x),collapse="-")})
contrast.matrix <- makeContrasts(contrasts = contrasts ,levels=design)

fit    = lmFit(resid,design)
fit2 <- contrasts.fit(fit, contrast.matrix)
fit2 <- eBayes(fit2)

for(j in 1:ncol(combos)){
    topSet = topTable(fit2, coef=j, number=nrow(fit2))

    # output or analyze top DE results here
}
```

2.3 Metabolite Analysis

We used a version of the same differential expression analysis to detect metabolites that were significantly different between statin-taking and non-statin-taking patients. Here, `metabolite_data` is a matrix of measured metabilte levels analagous to the `data` matrix used in step 1.1, and `info` is the same clinical information data frame used as input in steps 1.2 and 1.4. Metabolite data is first imputed (to fill in missing data and zeros) and normalized.

```
metabolite_data_imputed = apply(metabolite_data,2,function(s){
t = s
t[is.na(s)] = median(s,na.rm=T)
t
})

mins = apply(metabolite_data_imputed, 1,function(S){min(S[S!=0])})
for(i in 1:nrow(metabolite_data_imputed)){
    metabolite_data_imputed[i,metabolite_data_imputed[i,] == 0] = 0.9*mins[i]
}

metabolite_data_norm  = metabolite_data_imputed
for(i in 1:nrow(metabolite_data_imputed)){
    metabolite_data_norm[i,] = scale(metabolite_data_imputed[i,],
                                     center = T, scale = T)
}

design = model.matrix(~clinical_info_de$LipidLowerer)
fit    = lmFit(metabolite_data_de_norm,design)
fit2 <- eBayes(fit)

topSet = topTable(fit2, number=nrow(fit2))

# output or analyze top differential metabolites here
```

3 GO and KEGG Analysis

GO and KEGG annotations were retrieved using the `goseq` package, and enrichment analysis on these annotated was performed using the `topGO` package, both available on Bioconductor. Here, `allGenes` is a vector containing the Ensembl Gene IDs of all genes expressed in the tissue, and `DEGenes` is the list of differentially expressed genes from any of the differential expression analyses from step 2.

```
gene.map = getgo(allGenes,'hg19','ensGene')

a = rep(0, length(allGenes))
names(a) = allGenes
a[allGenes %in% DEGenes] = 1

ips = new("topGOdata", description = "Enrichment from DE",
    ontology = c("BP", "MF", "CC", "KEGG"),
    allGenes = allGenes, geneSel = names(a[a==1]),
    nodeSize = 10,
    annot = annFUN.gene2GO,
```

```
    gene2GO = gene.map)

test.stat = new("classicCount", testStatistic = GOFisherTest, name = "Fisher test")
res.fisher = getSigGroups(ips, test.stat)
res.final = GenTable(ips, classic = res.fisher, topNodes=500)
res.final$classic[res.final$classic == "<1e-30"] = 1/(1+1e-30)
res.final$classic = as.numeric(res.final$classic)
res.final = res.final[res.final$classic <= 0.1,]

# output or analyze GO enrichment results here
```

4 Coexpression Analysis

We performed coexpression analysis using the `coexpp` package, available at `https://bitbucket.org/multiscale/coexpp/`, and the `WGCNA` package, available at `https://horvath.genetics.ucla.edu/html/CoexpressionNetwork/Rpackages/WGCNA/`. Here, `data` is a data frame of expression data, either derived from the `resid` matrix from step 1.4 or downloaded from GTEx, reformatted and preprocessed for WGCNA. See `https://horvath.genetics.ucla.edu/html/CoexpressionNetwork/Rpackages/WGCNA/Tutorials/` for information on preprocessing steps required for WGCNA. Other than expression data, the code below takes two parameters: `cutHeight` and `beta`, the values of which were different by tissue as follows:

tissue	cutHeight	beta
LIV	200	NULL
AOR	225	NULL
Blood	250	NULL
VAF	250	6
MAM	250	NULL
SF	250	NULL
SKLM	200	7

```
sampleTree = flashClust(dist(data), method = "complete")
clust = cutreeStatic(sampleTree, cutHeight = cutHeight, minSize = 5)
keepSamples = (clust==1)
samplesRemoved = data[keepSamples, ]

coex <-coexpressionAnalysis(as.matrix(samplesRemoved),
                            cut="tree",beta = beta)

# output or analyze coexpression results here
```

5 eQTL analysis

eQTL analysis was performed with the standalone tools fastQTL and MetaXcan; see Methods and the documentation of these tools: `http://fastqtl.sourceforge.net/` and `https://github.com/hakyimlab/MetaXcan`.

6 Bayesian Network Analysis

6.1 Causal Inference Test

To seed the multiscale network with prior edges, we performed a causal inference test using the `citpp` package, available at `https://bitbucket.org/multiscale/coexpp`. The main input data are 3 matrices: `SNPs`, whose entries are SNP dosages (0, 1, or 2 for genotyped SNPs, a number between 0 and 2 for imputed SNPs); `met_data`, whose entries are measured metabolite levels; and `gene_data`, whose entries are measured gene expression levels. For all three, rows are samples and columns are measurements. This step also requires a data frame, `eQTL_data`, where rows are all possible combinations of metabolites and genes, and columns contain information about that metabolite, that gene, and the eQTL for that gene. In particular, the columns `snp_Idx`, `gene_Idx`, and `met_Idx` are the indices of the eQTL, gene, and metabolite in the input matrices; and `SNP_Met_pval` is the p-value of association between the eQTL and the metabolite.

```
L = as.matrix(SNP_expr)
G = as.matrix(gene_data)
T = as.matrix(met_data)
trios = as.matrix(cbind(eQTL_data$snp_Idx,eQTL_data$gene_Idx,eQTL_data$met_Idx))
cit_causal = cit(L,G,T, trios,threads=10)
cit_reactive = cit(L,T,G, trios[,c(1,3,2)],threads=10)

colnames(cit_reactive) = paste(colnames(cit_reactive),"_reactive",sep="")

res = cbind(eQTL_data,cit_causal,cit_reactive)
load("/sc/orga/projects/STARNET/ariella/cis_trans_causlity/rerun/geneNames_all.Rdata")

res$causal = res$p_cit <= 0.05 & res$p_cit_reactive > 0.05 & res$SNP_Met_pval <= 0.05 & res$
res$reactive = res$p_cit > 0.05 & res$p_cit_reactive <= 0.05 & res$SNP_Met_pval <= 0.05 & re

# output or process results data frame here
```

6.2 GLD Module Expansion

We expanded the GLD module using PEXA, which is a standalone program executed in Java. Please see the original PEXA publication (`https://dx.doi.org/10.1101/gr.087890.108`) or contact its authors for details on how to run PEXA.

6.3 Network Construction

We ran RIMBANET using the `run_BN.sh` wrapper, available at `https://bitbucket.org/multiscale/run_bn/`. No input parameters are required beyond the data produced by previous steps. See the documentation there.

7 Key Driver Analysis

We ran key driver analysis using the `keyDriver` package, available at `https://github.com/kippjohnson/RASNetwork/tree/master/Code/RPackages/keyDriver`. This package is invoked from the command line using the `R-keydriver-analysis.R` script, included in that repository. Edit that script to load the network data and set parameters; the parameters we used in this analysis were:

```
directed <- TRUE
layer <- 8
minDsCut <- 1
```
