# Supplementary material for: An integrative multiomic network model links lipid metabolism to glucose regulation in coronary artery disease
Source: Nat Commun. 2021 Jan 22;12:547. doi: 10.1038/s41467-020-20750-8 (PMC7822923; doi:10.1038/s41467-020-20750-8)
Supplement: Supplementary file 3 — Description of Additional Supplementary Files [file 41467_2020_20750_MOESM3_ESM.pdf]

Description of Additional Supplementary Files

File Name: Supplementary Data 1
Description: Glucose and lipid trait correlated DE signatures for all genes in all seven tissues, expressed as FDR.

File Name: Supplementary Data 2
Description: Gene Ontology for all differentially expressed gene lists in all seven tissues.

File Name: Supplementary Data 3
Description: Modules identified by WGCNA in all seven tissues.

File Name: Supplementary Data 4
Description: Correlation statistics between the first 3 principal components of the 20 glucose and lipid enriched modules and the glucose and lipid traits. P-values are from a two-sided Student's t test testing whether the correlation coefficient is zero. No multiple test correction was applied. FDR values are from the same test with a Benjamini-Hochberg correction applied.

File Name: Supplementary Data 5
Description: Results from molecular signature database (mSigDB) enrichment with GLD module as well as full Gene Ontology results. Only reporting results with an FDR<=5%.

File Name: Supplementary Data 6
Description: Correlation statistics between the 1st PCs of the GLD equivalent modules and the glucose and lipid traits in cohorts split by statins and HbA1c. P-values are from a two-sided Student's t test testing whether the correlation coefficient is zero. No multiple test correction was applied. FDR values are from the same test with a Benjamini-Hochberg correction applied.

File Name: Supplementary Data 7
Description: Significantly different metabolites in relation to statin status as well as metabolites correlation to GLD module. P-values are output from the edgeR R package's glmFit procedure, which internally uses a likelihood ratio test. No multiple test correction was applied. FDR values are from the same test with a Benjamini-Hochberg correction applied.

File Name: Supplementary Data 8
Description: Overlap between modules in STARNET liver and modules in the obese cohort.

File Name: Supplementary Data 9
Description: Results from MetaXcan analysis. P-values are output from MetaXcan, which internally uses a two-sided Wald test. No multiple test correction was applied.

File Name: Supplementary Data 10
Description: Archive containing Cytoscape network definition files (.cys) describing the four Bayesian Networks constructed from the STARNET data.

File Name: Supplementary Data 11
Description: Full key driver results from all 4 STARNET BNs.
