# Supplementary material for: An integrative multiomic network model links lipid metabolism to glucose regulation in coronary artery disease
Source: Nat Commun. 2021 Jan 22;12:547. doi: 10.1038/s41467-020-20750-8 (PMC7822923; doi:10.1038/s41467-020-20750-8)
Supplement: Supplementary file 15 — Reporting Summary [file 41467_2020_20750_MOESM15_ESM.pdf]

nature research

Corresponding author(s): Eric E Schadt, Johan LM Bjorkegren

Last updated by author(s): Aug 31, 2020

Reporting Summary

Nature Research wishes to improve the reproducibility of the work that we publish. This form provides structure for consistency and transparency in reporting. For further information on Nature Research policies, see our Editorial Policies and the Editorial Policy Checklist.

Statistics

For all statistical analyses, confirm that the following items are present in the figure legend, table legend, main text, or Methods section.

n/a	Confirmed	
☐	☒	The exact sample size (n) for each experimental group/condition, given as a discrete number and unit of measurement
☐	☒	A statement on whether measurements were taken from distinct samples or whether the same sample was measured repeatedly
☐	☒	The statistical test(s) used AND whether they are one- or two-sided<br>*Only common tests should be described solely by name; describe more complex techniques in the Methods section.*
☐	☒	A description of all covariates tested
☐	☒	A description of any assumptions or corrections, such as tests of normality and adjustment for multiple comparisons
☐	☒	A full description of the statistical parameters including central tendency (e.g. means) or other basic estimates (e.g. regression coefficient) AND variation (e.g. standard deviation) or associated estimates of uncertainty (e.g. confidence intervals)
☐	☒	For null hypothesis testing, the test statistic (e.g. F, t, r) with confidence intervals, effect sizes, degrees of freedom and P value noted<br>*Give P values as exact values whenever suitable.*
☒	☐	For Bayesian analysis, information on the choice of priors and Markov chain Monte Carlo settings
☒	☐	For hierarchical and complex designs, identification of the appropriate level for tests and full reporting of outcomes
☐	☒	Estimates of effect sizes (e.g. Cohen's d, Pearson's r), indicating how they were calculated

Our web collection on statistics for biologists contains articles on many of the points above.

Software and code

Policy information about availability of computer code

Data collection	No software was used in data collection.
Data analysis	The following publicly available software packages were used in data analysis: limma 3.21.1, edgeR 3.7.0, goseq 1.17.1, and topGO 2.17.0 in R 3.1.0; coexpp 0.1.0 and WGCNA 1.3.4 in R 3.1.1; MetaXcan 0.6.7; Matlab R2019a. The following previously published software packages are available on request from their original authors, as cited in the text: PEXA 1.0, RIMBANet 1.12, CIT 2.2, and KDA 0.1 .

For manuscripts utilizing custom algorithms or software that are central to the research but not yet described in published literature, software must be made available to editors and reviewers. We strongly encourage code deposition in a community repository (e.g. GitHub). See the Nature Research guidelines for submitting code & software for further information.

Data

Policy information about availability of data

All manuscripts must include a data availability statement. This statement should provide the following information, where applicable:

- Accession codes, unique identifiers, or web links for publicly available datasets
- A list of figures that have associated raw data
- A description of any restrictions on data availability

Data from the STARNET study are available through the Database of Genotypes and Phenotypes (dbGaP) under accession phs001203.v1.p1 (https://www.ncbi.nlm.nih.gov/projects/gap/cgi-bin/study.cgi?study_id=phs001203.v1.p1). Data from the mouse study are available through the Gene Expression Omnibus (GEO) under accession GSE157223 (https://www.ncbi.nlm.nih.gov/geo/query/acc.cgi?acc=GSE157223). Data from the Molecular Signatures Database (MSigDB) are available at https://www.gsea-msigdb.org/gsea/msigdb. Data from GTeX are available at https://gtexportal.org/. Data shown in Fig. 2a and 2c are included as Supplementary Data 1. Data shown in Fig. 2b are included as Supplementary Table 1. Data shown in Fig. 3a are included as Supplementary Data 7. Data shown in Figure 3b are included as Supplementary Data 6. Data shown in Fig. 4 are included as Supplementary Data 10.

Field-specific reporting

Please select the one below that is the best fit for your research. If you are not sure, read the appropriate sections before making your selection.

☒ Life sciences ☐ Behavioural & social sciences ☐ Ecological, evolutionary & environmental sciences

For a reference copy of the document with all sections, see nature.com/documents/nr-reporting-summary-flat.pdf

Life sciences study design

All studies must disclose on these points even when the disclosure is negative.

Sample size	For the mouse experiment, we grew 12 mice in each condition, as the maximum amount supported by the study's budget. 1 mouse in the LSS inhibition group did not produce enough data for analysis, so we dropped that mouse, as well as one mouse chosen at random from the control group so the sample sizes would be equal. For all human data, we used only pre-existing data and determined sample size by using all available samples.
Data exclusions	For each tissue, transcripts having less than 1 count per million in at least 10% of samples were removed. Additionally, within each tissue, samples that differed from the mean by more than 3 standard deviations in the first 2 principal components calculated by PCA across all transcripts were identified as outliers and removed. Both of these steps were intended to remove technical artifacts. These criteria were decided on before examining the results of the analysis. These exclusions were applied to all analyses involving human transcriptome data.
Replication	We replicated the presence of the GLD module in three independent datasets: a separate cohort of obese human patients, the Hybrid Mouse Diversity Panel (HMDP), and the Genotype-Tissue Expression (GTEx) project. We also validated the effect of the GLD module on blood glucose levels in an in vivo mouse experiment. All attempts at replication were successful.
Randomization	Randomization is not relevant to this study, since it is based on computational analysis of previously collected data and did not involve dividing participants into groups.
Blinding	Blinding is not relevant to this study, since it is based on computational analysis of previously collected data and did not involve dividing participants into groups.

Reporting for specific materials, systems and methods

We require information from authors about some types of materials, experimental systems and methods used in many studies. Here, indicate whether each material, system or method listed is relevant to your study. If you are not sure if a list item applies to your research, read the appropriate section before selecting a response.

Materials & experimental systems

n/a	Involved in the study	
☒	☐	Antibodies
☒	☐	Eukaryotic cell lines
☒	☐	Palaeontology and archaeology
☐	☒	Animals and other organisms
☒	☐	Human research participants
☒	☐	Clinical data
☒	☐	Dual use research of concern

Methods

n/a	Involved in the study	
☒	☐	ChIP-seq
☒	☐	Flow cytometry
☒	☐	MRI-based neuroimaging

Animals and other organisms

Policy information about studies involving animals; ARRIVE guidelines recommended for reporting animal research

Laboratory animals	Six-week-old female C57BL/6J (B6) mice obtained from The Jackson Laborat.ry (Bar Harbor, ME) . Animals were kept at 73 degrees Fahrenheit and 40% humidity.
Wild animals	The study did not involve wild animals.
Field-collected samples	The study did not involve field-collected samples.
Ethics oversight	Mice were maintained in accordance with University of California, Los Angeles Institution Animal Care and Use Committee protocols.

Note that full information on the approval of the study protocol must also be provided in the manuscript.
